# Forecasting the COVID-19 Pandemic in Saudi Arabia Using a Modified Singular Spectrum Analysis Approach: Model Development and Data Analysis

**DOI:** 10.2196/21044

**Published:** 2021-03-31

**Authors:** Nader Alharbi

**Affiliations:** 1 King Saud bin Abdulaziz University for Health Sciences King Abdullah International Medical Research Center Riyadh Saudi Arabia

**Keywords:** COVID-19, prediction, singular spectrum analysis, separability, eigenvalues, Saudi Arabia

## Abstract

**Background:**

Infectious disease is one of the main issues that threatens human health worldwide. The 2019 outbreak of the new coronavirus SARS-CoV-2, which causes the disease COVID-19, has become a serious global pandemic. Many attempts have been made to forecast the spread of the disease using various methods, including time series models. Among the attempts to model the pandemic, to the best of our knowledge, no studies have used the singular spectrum analysis (SSA) technique to forecast confirmed cases.

**Objective:**

The primary objective of this paper is to construct a reliable, robust, and interpretable model for describing, decomposing, and forecasting the number of confirmed cases of COVID-19 and predicting the peak of the pandemic in Saudi Arabia.

**Methods:**

A modified singular spectrum analysis (SSA) approach was applied for the analysis of the COVID-19 pandemic in Saudi Arabia. We proposed this approach and developed it in our previous studies regarding the separability and grouping steps in SSA, which play important roles in reconstruction and forecasting. The modified SSA approach mainly enables us to identify the number of interpretable components required for separability, signal extraction, and noise reduction. The approach was examined using different levels of simulated and real data with different structures and signal-to-noise ratios. In this study, we examined the capability of the approach to analyze COVID-19 data. We then used vector SSA to predict new data points and the peak of the pandemic in Saudi Arabia.

**Results:**

In the first stage, the confirmed daily cases on the first 42 days (March 02 to April 12, 2020) were used and analyzed to identify the value of the number of required eigenvalues (*r*) for separability between noise and signal. After obtaining the value of *r*, which was 2, and extracting the signals, vector SSA was used to predict and determine the pandemic peak. In the second stage, we updated the data and included 81 daily case values. We used the same window length and number of eigenvalues for reconstruction and forecasting of the points 90 days ahead. The results of both forecasting scenarios indicated that the peak would occur around the end of May or June 2020 and that the crisis would end between the end of June and the middle of August 2020, with a total number of infected people of approximately 330,000.

**Conclusions:**

Our results confirm the impressive performance of modified SSA in analyzing COVID-19 data and selecting the value of *r* for identifying the signal subspace from a noisy time series and then making a reliable prediction of daily confirmed cases using the vector SSA method.

## Introduction

One of the main issues that threatens human health worldwide is infectious diseases. Recently, the 2019 outbreak of the new coronavirus, SARS-CoV-2, which causes the disease known as COVID-19, has led to a global pandemic [1,2]. The first case of the virus was recognized and reported on December 31, 2019, in the city of Wuhan, the capital of Hubei Province in China [3]. The virus then spread rapidly worldwide and has affected more than 200 countries [4].

The number of cases and deaths from SARS-CoV-2 globally are considered to be a serious problem [5,6]. As of May 12, 2020, the number of confirmed cases worldwide was more than 4 million, with approximately 200,000 deaths. Although the outbreak appears to have abated in China, the virus and its impact are still spreading globally, and the case numbers are increasing. This is leading to concerns about variations in the affected cases and the mortality rate of the pandemic. Furthermore, there is much concern about the global economic impact of the crisis. It is now understood that the devastating influence of the virus on the economy and world health is without precedent [7].

In addition, several urgent queries related to transmission dynamics, mitigation, and control measures of COVID-19 have been raised, and researchers are attempting to use mathematical modeling to answer these important questions [8]. For example, the containment of transmission, plans such as quarantine, social distancing, and contact tracing of infected or suspected carriers, and lockdowns in regions or countries to address the disease have been included in the results of model predictions [9,10].

There are several standard epidemiological models for modelling epidemics, such as the susceptible, infectious, recovered (SIR) model [11-13]. Many studies have been conducted to model the pandemic using various methods, such as deep learning-based models [14], a simple iteration method [15], generalized additive models [16], which were used to estimate the three parameters of time-dependent transmission, time-dependent recovery, and time-dependent death rates from the outbreak; also, a hybrid model including 2D curvelet transformation, the chaotic salp swarm algorithm, and a deep learning technique was used to identify people infected with SARS-CoV-2 from x-ray images [17].

The primary objective of this study is the construction of a reliable, robust, and interpretable model for describing, decomposing, and forecasting the number of confirmed COVID-19 cases and predicting the peak of the pandemic in Saudi Arabia. The rate of mortality in Saudi Arabia is low, less than 1% at the time of writing this paper (May 12, 2020). Therefore, we were only interested in new daily cases of people affected by SARS-CoV-2 in an attempt to detect its peak. The number of cumulative cases was more than 40,000 as of May 12, 2020.

Because our aim was to analyze the daily data series of COVID-19, we sought to use a promising, reliable, and capable method for analyzing time series. A number of methods can be used to perform such an analysis; however, several of these methods are parametric and thus have requirements such as linearity or nonlinearity of a particular form.

An alternative method is to use nonparametric approaches that are neutral with respect to problematic areas of specification, such as linearity, stationarity, and normality [18]. These approaches can represent a reliable and superior means of decomposing time series data. Singular spectrum analysis (SSA) is a relatively new nonparametric technique that has been proved to be effective in several time series applications in different disciplines, such as genetics and biology [19,20], medicine [21,22], engineering [23,24], and economics and finance [25,26]. For the history of SSA, see [27,28], and for more details on the theory of SSA and its applications, refer to [29,30]. A comprehensive review of the SSA method and descriptions of its extensions and modifications can be found in [31].

The SSA technique is considered to be a useful tool that can be applied to solve many problems, such as smoothing; finding trends in different resolutions; simultaneous extraction of cycles with small and large periods; extraction of seasonality components; extraction of periodicities with varying amplitudes; and simultaneous extraction of complex trends and periodicities [30]. It should be noted that SSA is not linked with generalized autoregressive conditional heteroskedasticity, advanced autoregressive integrated moving average, wavelets, or other methods of this type. However, it has close links with certain methods of multivariate statistics and with signal methods such as projection pursuit and principal component analysis [30,32,33].

Although signals can be affected by internal or external noise, which often has unknown characteristics, they can be identified if the signal and noise subspaces are accurately separated. It is known that removing noise from any signal is necessary for analyzing any time series and is helpful in properly decomposing signals [34].

The main idea of SSA is to analyze the main series into different components, then reconstruct the noise-free series for further analysis. This process depends upon two main choices: the window length *L* and the number of required eigenvalues, denoted by *r*, for reconstruction. Therefore, appropriate selection of *L* and *r* leads to perfect analysis and separability between the time series components. It was discussed in [35] that for a series of length *N*, selecting *L*=*N/*4 is common practice. It should also be mentioned that *L* needs to be sufficiently large but no larger than half of the series [29]. In [36], it was shown that for a series of length *N* and the optimal selection of the number of eigenvalues *r* for reconstructing the signal, the appropriate value of the window length is *median*{1*, …, N*}. Although various attempts have been made, no universal rule has been established for obtaining optimal selections of *L* and *r*.

We proposed an approach in [37-39] for the selection of the value of *r* for noise reduction, filtering, and signal extraction in SSA. This approach has also been applied to the distinction of noise from chaos in time series analysis [40] and for the correction of noise in gene expression data [41]. In [39], we developed the approach and introduced new criteria to the discrimination between epileptic seizure and normal electroencephalogram (EEG) signals, the filtering of the EEG signal segments, and elimination of the noise included in the signal. The approach is mainly used to identify the required number of eigenvalues or singular values corresponding to the signal component, which depends on the distribution of the eigenvalues of a scaled Hankel matrix. The correlation between eigenvalues, the coefficients of skewness, the kurtosis, and the variation of the distribution of the eigenvalues were proposed and proved to be new criteria for the separability between the signal and noise components, as they can split the eigenvalues into two groups [38]. Different simulated and real signals were used to consider different signal-to-noise (SNR) ratios in [38,39] and were evaluated to show the ability of the approach in the selection of *r*.

The remainder of this paper is structured as follows. The Methods section gives a short description of the modified SSA approach and its algorithm. In the Results section, we show that this approach can be used to decompose synthetic data into two main distinct subspaces, and we then discuss the implementation of the approach in decomposing and reconstructing series of COVID-19 daily cases. This section also presents the forecasting of the COVID-19 pandemic in Saudi Arabia using vector singular spectrum analysis (VSSA) of the signal extracted by modified SSA. The Discussion section draws the conclusion of the paper and suggests ideas for future work.

## Methods

### The Modified SSA Method: Review

This section presents a short description of the modified SSA used in this manuscript (for more details, refer to [38]). A time series was decomposed by the technique into a sum of components, allowing for identification of each as either a main or noise component. The goal was to consider the signal as a whole so that we could identify the appropriate value of *r* related to the whole signal component. In other words, we were not interested in each signal component; thus, the selection of *L* rational to the periodicity of the signal components was less important [30]. Therefore, the modified SSA method focused on the selection of *r* to identify the signal subspace.

Consider a one-dimensional series *Y_N_* = (*y*_1_, …, *y_N_*) of length *N*. Transferring this series into a multidimensional series *X*_1_, …, *X_K_ ,* where *X_i_* = (*y*_1_, …, *y_i_*_+_*_L_*_–1_)*^T^* ∈ ***R****^L^* provides 
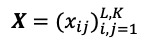
, where *L* is an integer (2 ≤ *L* ≤ *N*/2) and *K = N – L* + 1.

A matrix ***X*** is a Hankel matrix, in which all the elements along the diagonal *I* + *j* = *const* are equal. Set ***B*** = ***XX****^T^*, denote by *λ_i_* (*i* = 1, …, *L*) the eigenvalues of ***B*** taken in decreasing order of magnitude (*λ_1_ ≥* ⋯ *λ_L_* ≥ 0), and denote by *U*_1_, …, *U_L_* the orthonormal system of the eigenvectors of matrix ***B*** corresponding to these eigenvalues. The singular value decomposition (SVD) of matrix **X** can be written as follows:

***X*** = ***X***_1_ + ⋅⋅⋅ + ***X****_L_*
**              (1)**

where 
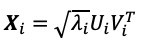
. The elementary matrices ***X****_i_* having rank 1, ***U****_i_*, and ***V****_i_* are the left and right eigenvectors of matrix ***X***. Note that the collection 
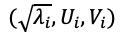
 is called the *i^th^* eigentriple of the SVD. Note also that 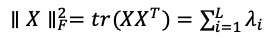 and 
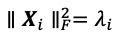
, where ║ ║*_F_* denotes the Frobenius norm.

Fundamental to the question of eigenvalue behavior, *λ_i_*, is that if the series size increases, there is a corresponding increase in the eigenvalues. This problem can be overcome if ***B*** is divided by its trace, ***A = B***/*tr*(***B***), which provides several important properties [37]. Let ζ_1_, …, ζ*_L_* denote the matrix ***B*** eigenvalues in decreasing order of magnitude (1 ≥ ζ_1_ ≥ ⋅⋅⋅ ζ*_L_* ≥ 0). The simulation is performed to obtain the distribution of ζ_1_ and to understand the behavior of each eigenvalue. This helps identify the value of *r*. Here, the goal was to establish the distribution and related forms of ζ_1_ that would be used to select the appropriate value of *r* for removing noise from the COVID-19 series.

It was proved in our previous work [38] that the largest eigenvalue has a positive skewed distribution for a white noise process. Therefore, if *skew*(ζ*_c_*) (*c* ∈ {1, …, *L*})is the maximum, and the pattern for *skew*(ζ*_c_*) to *skew*(ζ*_L_*) has the same pattern, the same as that which emerged for the white noise, then the first *r* = *c* 1 eigenvalues correspond to the signal and the remaining eigenvalues correspond to the noise. A similar procedure can be performed using the coefficients of kurtosis and the variation of ζ*_i_*. Furthermore, if *ρ_S_*(ζ*_c_*_–1_, ζ*_c_*) is the minimum, and the pattern for the set 
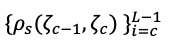
 is similar to what was observed for the white noise, then we select the first *r* eigenvalues for the signal and the remainder for the noise component (for more information, see [38]).

In this research, we used the third and fourth central measure moments of the distribution, which are the skewness (*skew*) and kurtosis (*kurt*). Skewness is a measure of asymmetry of the data distribution, while kurtosis describes the distribution of observed data in terms of shape or peak. We used these measures as criteria for choosing the value of *r*, which can be calculated for a simulation *m* as follows:

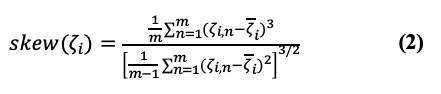

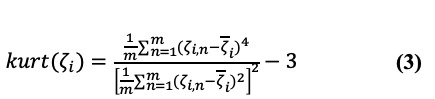





Moreover, the coefficient of variation (*CV*), which is defined as the ratio of the standard deviation *σ*(ζ*_i_*) and 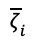, can be calculated mathematically from the following formula:

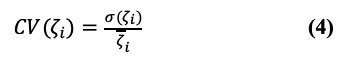



In addition, the Spearman correlation *ρ_S_* between the eigenvalues ζ*_i_* and ζ*_j_* (*i*, *j* = 1, …, *L*) was calculated to enhance the results obtained by those measures:

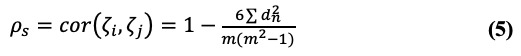



where *d_n_* = *x_n_* – *y_n_* (*n* = 1, …, *m*) is the difference between *x_n_* and *y_n_,* which are the ranks of ζ*_i_* and ζ*_j_*, respectively, and ζ*_i,n_* is the *n*-th observation for the *i*-th eigenvalue (ζ*_j_*), 
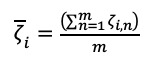
.

These measures of difference between the eigenvalues related to the signal and noise components can specify the cutoff point of separability, namely, the number of leading SVD components that are separated from the residual. Therefore, the final cutoff point of separability between the signal and noise components obtained by the suggested measures corresponds to the rank estimation.

The eigenvalues can be split into two groups by using the above criteria; the first group corresponds to the signal, and the second corresponds to the noise component. Furthermore, the Spearman correlation *ρ_S_* between ζ*_i_* and ζ*_j_* was calculated to support the outcomes obtained by those measures. The absolute value of the correlation coefficient was considered; 1 shows that ζ*_i_* and ζ*_j_* have a perfect positive correlation, while 0 indicates there is no correlation between them. The matrix of the absolute values of the Spearman correlation gives a full analysis of the trajectory matrix, and in this analysis, each eigenvalue corresponds to an elementary matrix of the SVD. Note that if the absolute value of *ρ_S_* is close to 0, the corresponding components are almost orthogonal; however, if it is close to 1, the two components are far from being orthogonal, and thus it is difficult to separate them. Therefore, if *ρ_S_*=0 between two reconstructed components, these two reconstructed series are separable. The results of *ρ_S_* between the eigenvalues for the white noise are quite large (see [38]), which aids the discrimination of the noise part.

Once *r* is identified, the matrices ***X****_i_* can be split into two groups. Therefore, Equation 1 can be written as

***X*** = ***S*** + ***E*              (6)**

where 
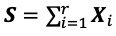
 is the signal matrix and 
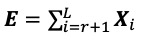
 is the noise matrix. We then use diagonal averaging to transform matrix ***S*** into a new series of size *N* (see [29]).

### The Algorithm

The algorithm consisted of two main stages. The steps in the first stage used the coefficients of skewness, kurtosis, variation, and correlation to help obtain the optimal value of *r* for the separability between signal and noise, as these coefficients split the eigenvalues into two groups. The steps in the second stage were used to reconstruct the free noise series.

The steps in Stage 1 are outlined below:

1. Map a one-dimensional time series *Y_N_* = *y*_1_, …, *y_N_* into s multidimensional series *X*_1_, …, *X_K_* with vectors *X_i_* = (*y_i_*, …, *y_i_*_+_*_L_*_–1_) ∈ ***R****^L^*, where the window length *L* is an integer; 2 ≤ *L* ≤ *N*/2, and *K* = *N* – *L* + 1. This step gives us the Hankel matrix 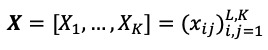.
2. Compute the matrix ***A*** = ***XX****^T^*/*tr*(***XX****^T^*).
3. Decompose matrix ***A*** as ***A*** = ***PΓP****^T^*, where ***Γ*** = *diag*(ζ*_i_*, …, ζ*_L_*) is the diagonal matrix of the eigenvalues of ***A*** that has the order (1 ≥ ζ*_i_*, …, ζ*_L_* ≥ 0) and *P* = *P*_1_, …, *P_L_* is an orthogonal matrix whose columns are the corresponding eigenvectors.
4. Simulate the original series *m* times and calculate the eigenvalues for each series. We simulate *y_i_* from a uniform distribution with boundaries *y_i_* – *a* and *y_i_* – *b*, where *a* = |*y_i_*_–1_ – *y_i_*| and *b* = |*y_i_* – *y_i_*_+1_|.
5. Compute the skewness coefficient for each eigenvalue, *skew*(ζ*_i_*). If *skew*(ζ*_c_*) is the maximum, and the pattern for *skew*(ζ*_c_*) to *skew*(ζ*_L_*) has a similar pattern to that of the white noise, select *r* = *c* – 1.
6. Compute the coefficient of kurtosis for each eigenvalue, *kurt*(ζ*_i_*). If *skew*(ζ*_c_*) is the maximum, select *r* = *c* – 1.
7. Compute the coefficient of variation, *CV*=ζ*_i_*. The result of the *CV* splits the eigenvalues into two groups; the eigenvalues from ζ*_i_* to ζ*_c_*_–1_ correspond to the signal, and the remaining eigenvalues, which have an almost U shape, correspond to the noise.
8. Compute the absolute values of the correlation matrix between the eigenvalues and represent them in a 20-grade grey scale from white to black corresponding to the values of the correlations from 0 to 1. This matrix also splits the eigenvalues into two groups; the eigenvalues from ζ*_i_* to ζ*_r_* correspond to the signal, and the remaining eigenvalues correspond to the noise.

The steps in Stage 2 are outlined below:

1. Calculate the approximated signal matrix 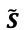, that is, 
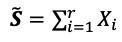
, where *r* is obtained from the first stage, and 
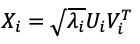
, where *U_i_* and *V_i_* represent the left and right eigenvectors of the trajectory matrix, respectively.
2. Averaging over the diagonals of the matrix 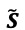 gives a one-dimensional series, which is the approximate signal 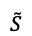.

The capabilities of modified SSA using different types of synthetic data, including series generated from chaotic map systems with different SNR ratios, are presented in [38]. This study confirms that the approach works promisingly for any series that is mixed with a low or high noise level.

Each eigenvalue or singular value contributes to the trajectory matrix decomposition. We can consider the ratio to be the characteristic of matrix *H_i_* to Equation 1. Therefore, 
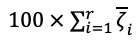
 is considered to be characteristic of the optimal approximation of ***H*** by matrices of rank *r.*

## Results

### Separability in Synthetic Data

It should be noted that using the standard criteria in basic SSA, the weighted correlation (*w*-correlation) for separability and grouping (for more information, see [29]), does not always provide good separability and correct selection of *r*, especially for real data.

It was shown in [38] that the results based on *skew*, *kurt*, *CV*, and *ρ_S_* are more accurate than those obtained by the *w*-correlations for small window lengths, particularly for data in which a linear trend is included in the series.

We therefore used modified SSA—in particular, some of the proven criteria on the distribution of ζ*_i_*, as given in the previous sections—to identify *r*. The results were plausible and reliable.

Below, we provide a synthetic example to show the capability of the approach before applying it to the COVID-19 data; for more examples considering different types of series and evaluations with different criteria, refer to [38].

**Figure 1:**
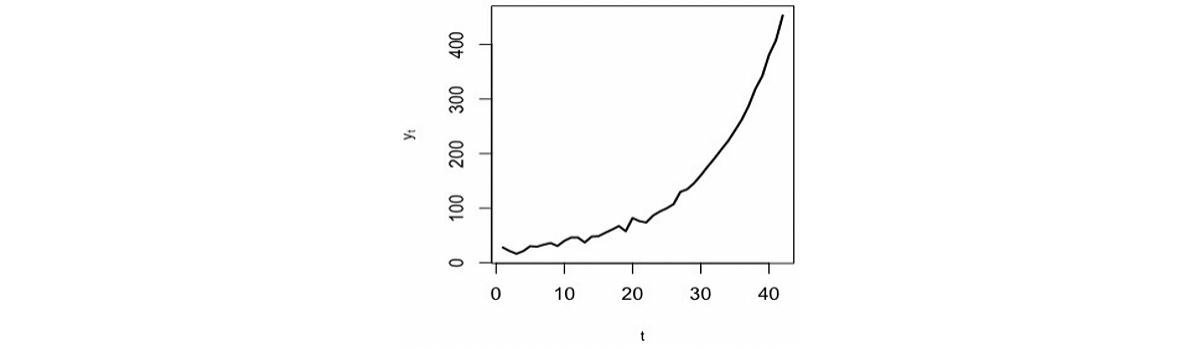
Realization of the simulated exponential trend series.

In the following example, a white noise process 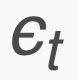 was added to an exponential trend series:

*Y_t_* = *α*_1_ + *α*_2_ exp(*α*_2_*t*) + 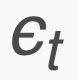               **(7)**

where *t*=(1, …, *N*), *N*=42, *α*_1_=10, *α*_2_=0.09, and 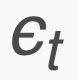 is a Gaussian white noise process with variance 1 (see Figure 1). It is obvious that the number of eigenvalues required to reconstruct the signal for this series is 2, as we have added a constant to the exponential curve, which corresponds to the rank estimation (see [29]).

**Figure 2:**
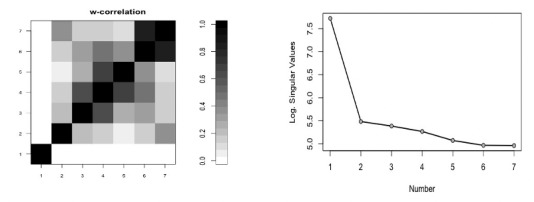
Left: w-correlation matrix for the seven reconstructed components of the simulated series. Right: logarithms of the seven eigenvalues of the simulated series. w-correlation: weighted correlation.

**Figure 3:**
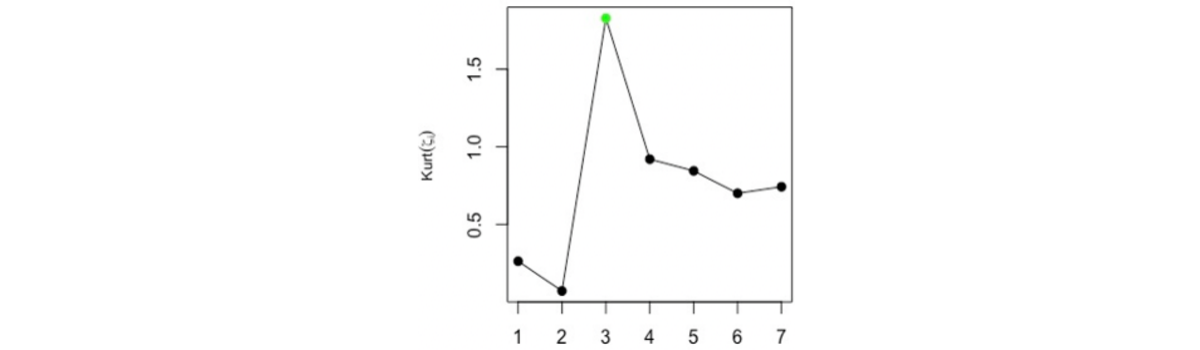
Kurt of ζ_*i*_ for the simulated series. Kurt: kurtosis.

**Figure 4:**
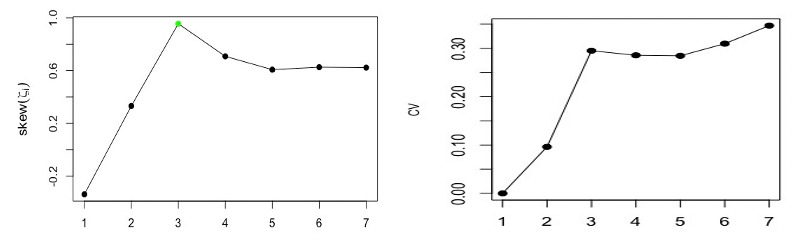
Left: skew of ζ_*i*_ for the simulated series. Right: CVs of ζ_*i*_ for the simulated series. CV: coefficient of variation; skew: skewness.

Based on observations of the *w*-correlations and the logarithm of the eigenvalues, one may use only the first component to extract the signal (see Figure 2). However, using the suggested measures and criteria gives the correct value of *r*. Figure 3 shows the kurtosis coefficient of ζ*_i_* (*i*=1, …, *L*). The maximum value of the kurtosis coefficient is considered as one of the rules and indicators used for the start of the noise. It is clear that the maximum kurtosis coefficient of ζ*_i_* is obtained for ζ*_c_*_=3_. Therefore, the number of eigenvalues required to extract the signal is *r* = *c* – 1 = 2. Similar results were obtained using the values of *skew* and *CV* (see Figure 4).

**Figure 5:**
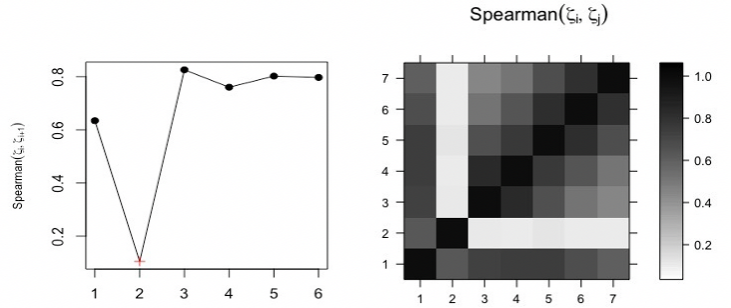
Left: Spearman correlation of (ζ_*i*_,ζ(_*i+1*_). Right: matrix of Spearman correlation between (ζ_*i*_,ζ_*j*_).

In addition, the Spearman correlation coefficient between ζ*_i_* and ζ*_i_*_+1_ was calculated; Figure 5 (left) shows the correlation between ζ*_i_* and ζ*_i_*_+1_. For the correlation coefficient, the minimum value of *ρ_S_* between ζ*_i_* and ζ*_i_*_+1_ was used as another indicator for the cutoff point. The results were similar to those that emerged using other criteria and confirmed that the approach works properly. Different criteria, such as root mean square error and mean absolute error, were used in [38] to evaluate the approach, and the results confirmed that the modified approach is a promising one.

The correlation matrix also enables us to distinguish and separate the different components from each other. Therefore, the correlation matrix of ζ*_i_* identifies the separability between the components. If the absolute value of the correlation coefficient between ζ*_i_* and ζ*_j_* is small, then the corresponding components are almost orthogonal; however, if the value is large, then the corresponding series are far from being orthogonal, and thus they are not neatly separable. It is clear that the signal can be separated from the noise, as the top right-hand pattern from the correlation matrix is related to the white noise process (see Figure 5, right).

### COVID-19 Data Analysis

**Figure 6:**
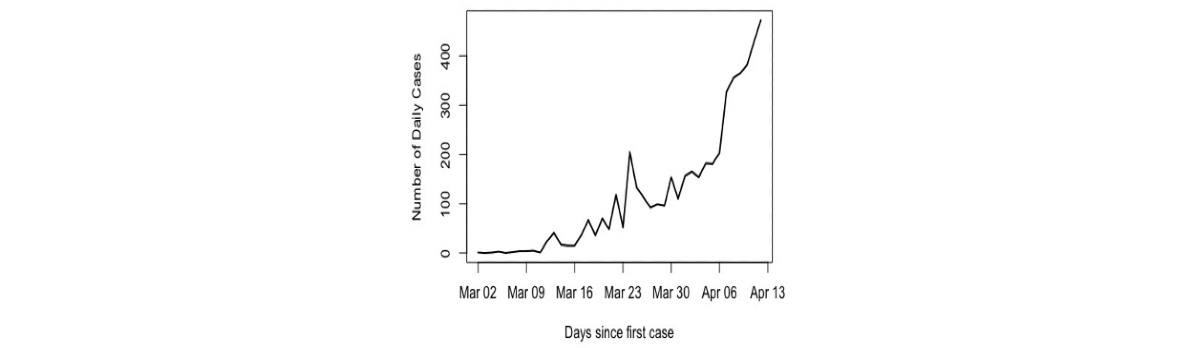
Time series of daily confirmed COVID-19 cases in Saudi Arabia (March 2 to April 12, 2020).

The daily numbers of confirmed cases of COVID-19 in Saudi Arabia [42] were used in this research. First, we used data from the first 42 days, from March 2 to April 12, 2020. The aim was to analyze the data, make predictions from April 13, 2020, and detect the peak. The number of daily cases series is shown in Figure 6. Second, we updated our data on May 20, 2020, to include values from April 13 to May 12, 2020; thus, the total became 81 values. This did not affect the required number of eigenvalues for the reconstruction stage, as will be discussed in the following section.

#### Separability and Selection of the Components

Starting with the first set of COVID-19 data, as mentioned earlier, because our aim was to extract the signal as a whole, we could choose any value for *L*, with the goal to find the best choice of *r*. Furthermore, in our previous research [38], we showed that it is possible to use a small window length when analyzing exponential series, like the series of COVID-19 cases. The selection of *L*=7 provided the best and most reasonable results with the required *r* that would be obtained by the proposed approach.

The results based on these measures in extracting the signal for forecasting gave a curve with a likely peak. However, the predictions using various other choices for *L* and *r* did not indicate any end or peak for the pandemic and in fact showed exponential increases; such increases are impossible, as the pandemic will not continue forever. This finding also supports the obtained results. Therefore, the next important task was the selection of the number of eigenvalues *r* required for the reconstruction and building of the model for forecasting.

**Figure 7:**
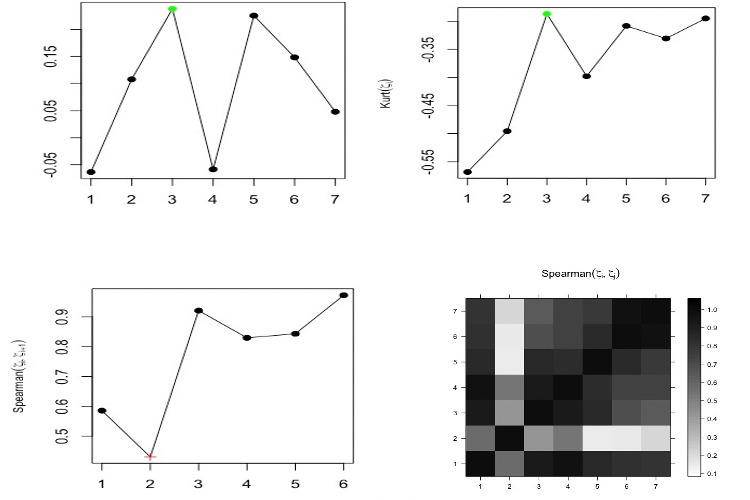
Coefficients of skewness (top left) and kurtosis (top right) for each eigenvalue and the correlations between ζ_*i*_ and ζ_*i+1*_ (bottom left) and the results of the matrix correlations (bottom right) for L=7.

Figure 7 illustrates the coefficients of skewness and kurtosis for each eigenvalue and the results of the matrix correlations and the correlations between ζ*_i_* and ζ*_i_*_+1_ for *L*=7. As shown by the results, for the COVID-19 daily series, the maximum values of *skew* and *kurt* are observed for ζ*_c_*_=3_, and the minimum value of *ρ_S_* is obtained between ζ*_c_*_–1=3_ and ζ*_c_*_=3_. In addition, the matrix of the Spearman correlation for ζ*_i_* and ζ*_j_* splits the eigenvalues or the components into two groups, which indicates that the value of *r* is 2.

**Figure 8:**
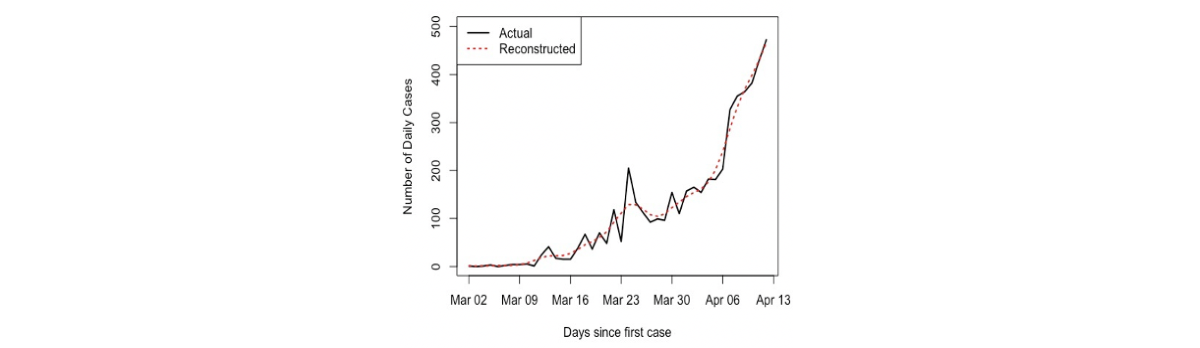
Plot of the first time series of daily COVID-19 cases in Saudi Arabia and the fitted curve.

Figure 8 shows the results of the reconstructed series obtained by using *L*=7 and eigentriples *r*=2. The red and black lines correspond to the reconstructed series and the original series, respectively. It appears that the reconstructed series that was obtained is good. However, it will be shown later that the reconstructed series using the whole data set is better than this fitted series.

#### Prediction of Daily Cases of COVID-19 Using VSSA

After obtaining the reconstructed series, the next aim was to predict the data for daily new cases from April 13 to August 2020. There are two main forecasting methods in SSA: VSSA (VSSA) and recurrent singular spectrum analysis (RSSA). The VSSA forecasting algorithm is the most widely used in SSA [29]. Generally, this method is more robust than RSSA, especially when a series contains outliers or when facing large shocks in the series [43]. Therefore, we focused on the use of the VSSA algorithm for forecasting in this research, as recommended in [18].

### Vector Forecasting Algorithm

To perform SSA forecasting, the basic requirement is that the series satisfies a linear recurrent formula (LRF). The series *Y_N_* = [*y*_1_, …, *y_N_*] satisfies an LRF of order *L* 1 if

*Y_t_* = *a*_1_*y_t_*_-1_ + *a*_2_*y*_2_ + ⋅⋅⋅ + a_L–1_y*_t_*_–_*_L_*_+1_, *t = L* + 1, …, *N* **(8)**

The coefficient vector *A* = *a*_1_, …, *a_L_*_–1_ is defined as follows:

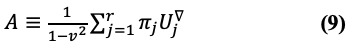



where 
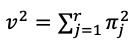
, 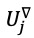 is the vector of the first *L* – 1 components of the eigenvector *U_j_*, and *π_j_* is the last component of *U_j_* (*j* = 1, …, *r*).

Consider the following matrix:

***Π = U***^∇^***U***^∇T^ + (1 – *v*^2^)***AA****^T^*
**              (10)**

Let us now define the linear operator:

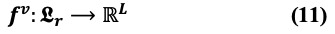



where 
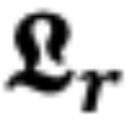
 = *span*{*U*_1_, …, *U_r_*} and

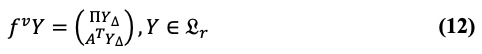



where *Y*_∆_ is the vector of the last *L* – 1elements of *Y_N_*. The vector *Z_j_* is defined as follows:

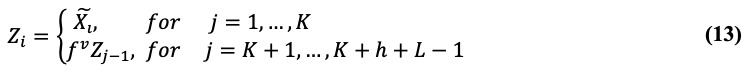



where 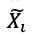 are the reconstructed columns of the trajectory matrix of the *i*-th series after grouping and leaving out noise components. Now, by constructing matrix ***Z*** = [*Z*_1_, …, *Z_K_*_+_*_h_*_+_*_L_*_–1_] and performing diagonal averaging, a new series 
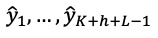
 is obtained, where 
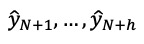
 from the *h* terms of the VSSA forecast.

As discussed above, the best values for reconstruction were *L*=7 and *r*=2. The values of *L*=6 and *r*=3 were the second-best choices based on the criteria presented earlier. For forecasting, the results of these two choices were compared by using the complement statistical test introduced in [44], which is proposed for distinguishing between the predictive accuracy of two sets of forecasts. It is a nonparametric test founded upon the principles of the Kolmogorov-Smirnov test and known as the KS predictive accuracy (KSPA) test. The test is useful for serving two different purposes. First, 2-sided KSPA is used to determine if there is a statistically significant difference between the distribution of forecast errors. Second, the 1-sided KSPA test exploits the principles of stochastic dominance to determine whether the forecasts with lower error also produce a stochastically smaller error than forecasts from a competing model, and it then allows for differentiation between the predictive accuracy of the forecasts [45].

The 2-sided KSPA test indicated that there was no statistically significant difference between the distribution of forecast errors at a 95% confidence level (*P*=.56). Moreover, there was insufficient evidence based on the one-sided KSPA test at the 5% significance level to conclude that the stochastic errors are different (*P*=.76). Therefore, the results confirm that there is no statistically significant difference between the two forecasts.

**Figure 9:**
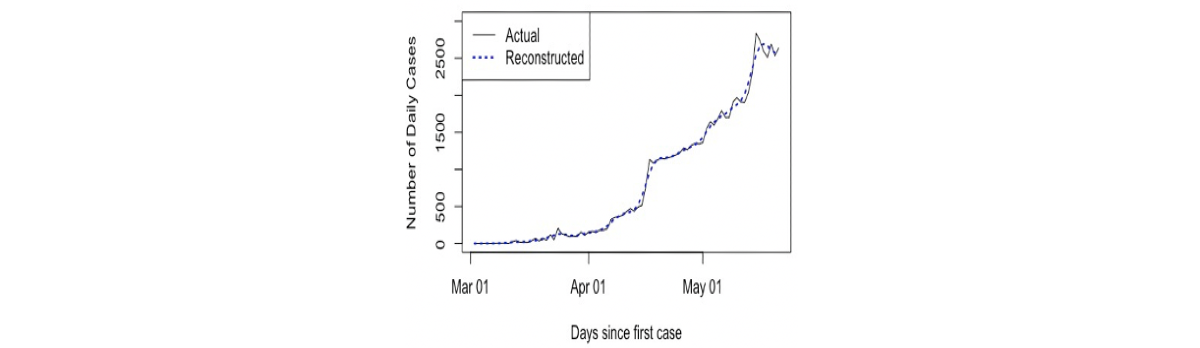
Plot of the entire time series of daily COVID-19 cases in Saudi Arabia and the fitted curve.

**Figure 10:**
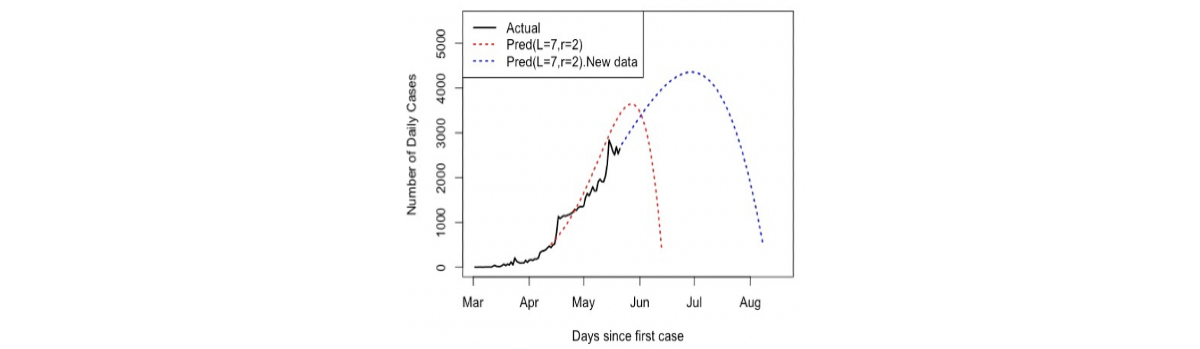
Comparison of the two forecasting scenarios with actual observations. Pred: predicted.

Consequently, we also concentrated only on the best values obtained, *L*=7 and *r*=2, for forecasting. Similar procedures were followed for the new data updated on May 20, 2020. The same values of *L* and *r* were used to analyze the new data and also for predicting confirmed cases 3 months ahead. Figure 9 shows the updated data and the reconstructed series by the first two eigentriples. It is obvious that the reconstructed series was obtained precisely. Figure 10 shows the two curve predictions and the overall actual data; the red curve is the prediction using the first set of data, and the blue curve is the prediction using the updated data set. It is clear that there is no great difference between the two curves, as the peak appears around the end of May in the red curve and toward the end of June in the blue curve, which was obtained using the updated data. In addition, the end of the pandemic is predicted to occur between July and the middle of August, with the total number of infected people at approximately 330,000.

## Discussion

A modified SSA approach was used in this research for the decomposition and forecasting of COVID-19 data in Saudi Arabia. The approach was examined in our previous research and was applied here to the analysis of COVID-19 data.

In the first stage, the first 42 values of confirmed daily cases (March 2 to April 12, 2020) were used and analyzed to identify the value of *r* for separability between the noise and signal. After obtaining the value of *r*, which was 2, and extracting the signals, VSSA was used for the prediction and determination of the pandemic peak. In the second stage, we updated the data and included 81 daily values. We used the same window length and number of eigenvalues for the reconstruction and forecasting of the points 90 days ahead. The results of both forecasting scenarios indicated that the peak would occur around the end of May or June and the crisis would end between the end of June and the middle of August 2020, with a total number of infected people of approximately 330,000.

All our results confirm the impressive performance of modified SSA in analyzing the COVID-19 data and selecting the value of *r* for identifying the signal subspace from a noisy time series, then making an accurate prediction using the VSSA method. Note that we did not examine all possible window length values in this research, and for forecasting, we only used basic VSSA.

In future research, we will include more data and consider different window lengths *L,* which may provide better forecasting. In addition, chaotic behavior in the COVID-19 data will be examined, as some of our results show strange patterns, as can be found in chaotic systems.

## Abbreviations

*CV*: coefficient of variation
EEG: electroencephalogram
KSPA: Kolmogorov-Smirnov predictive accuracy
*kurt*: kurtosis
RSSA: recurrent singular spectrum analysis
SIR: susceptible, infectious, recovered
*skew*: skewness
SNR: signal-to-noise ratio
SSA: singular spectrum analysis
SVD: singular value decomposition
VSSA: vector singular spectrum analysis
*w*-correlation: weighted correlation
